# Relation between intima-media thickness and bone mineral density in postmenopausal women: a cross-sectional study

**DOI:** 10.1590/S1516-31802011000300004

**Published:** 2011-05-05

**Authors:** Daniela Fodor, Cosmina Bondor, Adriana Albu, Laura Muntean, Siao-pin Simon, Laura Poanta, Alexandra Craciun

**Affiliations:** I MD, PhD. Rheumatologist and Associate Professor in Second Internal Medicine Clinic, "Iuliu Hatieganu" University of Medicine and Pharmacya, Cluj-Napoca, Romania.; II Statistician. Assistant Professor, Department of Medical Informatics and Biostatistics, "Iuliu Hatieganu" University of Medicine and Pharmacy, Cluj-Napoca, Romania.; III MD, PhD. Clinician and Associate Professor in Second Internal Medicine Clinic, "Iuliu Hatieganu" University of Medicine and Pharmacy, Cluj-Napoca, Romania.; IV MD. Rheumatologist and Assistant Professor in Rheumatology Clinic, "Iuliu Hatieganu" University of Medicine and Pharmacy, Cluj-Napoca, Romania.; V MD, PhD. Rheumatologist and Lecturer in Rheumatology Clinic, "Iuliu Hatieganu" University of Medicine and Pharmacy, Cluj-Napoca, Romania.; VI MD, PhD. Clinician and Lecturer in Second Internal Medicine Clinic, "Iuliu Hatieganu" University of Medicine and Pharmacy, Cluj-Napoca, Romania.; VII MD, PhD. Laboratory Medicine Specialist and Associate Professor, Department of Biochemistry, "Iuliu Hatieganu" University of Medicine and Pharmacy, Cluj-Napoca, Romania.

**Keywords:** Bone density, Carotid artery, common, Atherosclerosis, Osteoporosis, Postmenopause, Densidad ósea, Arteria carótida común, Aterosclerosis, Osteoporosis, Posmenopausia

## Abstract

**CONTEXT AND OBJECTIVES:**

Controversy exists regarding the relationship between atherosclerosis and osteoporosis. The aim of this study was to determine the relationship between intima-media thickness (IMT) of the common carotid artery (CCA), presence of calcified atherosclerotic plaques and bone mineral density (BMD) evaluated by dual energy X-ray absorptiometry (DXA), in postmenopausal women.

**DESIGN AND SETTING:**

Cross-sectional study at Second Internal Medicine Clinic, Cluj-Napoca, Romania.

**METHODS:**

We studied the IMT (left and right CCA and mean IMT) and T-score (lumbar spine L2-L4, femoral neck and total hip) in 100 postmenopausal women (mean age 64.5 years). The presence of calcified atherosclerotic plaque and osteoporotic vertebral fractures was also noted.

**RESULTS:**

IMT in the left and right CCA and mean IMT were significantly associated with T-score measured for the lumbar spine L2-L4, femoral neck and total hip, with lower T-score, in the osteoporotic group than in the normal and osteopenic groups (P < 0.05). IMT had a significantly negative correlation with the lumbar spine T-score and femoral neck T-score; and mean IMT with lowest T-score. Mean IMT (P < 0.001), high blood pressure (P = 0.005) and osteoporotic vertebral fractures (P = 0.048) showed statistical significance regarding the likelihood of developing atherosclerotic plaque.

**CONCLUSIONS:**

In women referred for routine osteoporosis screening, the relationship between CCA, atherosclerosis and osteoporosis can be demonstrated using either cortical or trabecular BMD. Vertebral fractures may be considered to be a likelihood factor for atherosclerotic plaque development.

## RESUMÉN

**CONTEXTO Y OBJETIVOS:**

Hay controversias en cuanto a la relación entre la arterioesclerosis y la osteoporosis. El objetivo del estudio fue determinar la relación entre el espesor mío-intimal (EMI) de la arteria carótida común (ACC), la presencia de placas arterioescleróticas calcificadas, y la densidad mineral ósea (DMO) evaluada a través de absorciometría de rayos X de energía dual (DEXA) en mujeres posmenopáusicas.

**DISEÑO Y ENTORNO:**

Estudio transversal en la Segunda Clínica de Medicina Interna, Cluj-Napoca, Rumania.

**MÉTODOS:**

Hemos estudiado el EMI (ACC izquierda y derecha y EMI promedio) y el T-score (espina lumbar L2-L4, cuello femoral y cadera total) en 100 mujeres posmenopáusicas (edad promedia 64.5 años). Se ha observado también la presencia de las placas arterioescleróticas calcificadas y de la fractura vertebral osteoporótica.

**RESULTADOS:**

El EMI en ACC izquierda y derecha, como también el EMI promedio se ha asociado sobre todo con el T-score medido en la espina lumbar L2-L4, cuello femoral y cadera total o el valor más bajo del T-score, en el grupo osteoporótico, en comparación con el grupo normal y el osteopénico (P < 0.05). Se ha registrado una correlación significativamente negativa entre el EMI y el T-score de espina lumbar, el T-score del cuello femoral, y el EMI promedio con el T-score más bajo. El EMI promedio (P < 0.001), la alta presión (P = 0.005), y las fracturas vertebrales osteoporóticas (P = 0.048) demostraron tener una importancia estadística con respecto a la probabilidad de formación de placas arterioescleróticas.

**CONCLUSIONES:**

En el caso de las mujeres sometidas a screening rutinario para la osteoporosis, la relación entre ACC, arterioesclerosis y osteoporosis se puede demostrar utilizando sea la DMO cortical o trabecular. Las fracturas vertebrales se pueden considerar como un factor de probabilidad para el desarrollo de las placas arterioescleróticas.

## INTRODUCTION

Osteoporosis and cardiovascular diseases are known to be major causes of morbidity and mortality in postmenopausal women.^1,2^ A large number of studies have demonstrated a relationship between bone pathology and vascular disease, thus suggesting that there are common pathways that affect negatively bone metabolism and the vasculature,^3-6^ and that the presence of one is a predictor for the other.^7^ However, the nature of the association between osteoporosis and atherosclerosis remains unknown.

Measurement of intima-media thickness (IMT) in the distal common carotid artery is increasingly being used as an independent risk factor for the development of cardiovascular events due to atherosclerosis.^8,9^ Carotid atherosclerosis has been associated with lumbar spine bone mass in postmenopausal women^10^ and calcification of the atherosclerotic plaques with low bone mineral density (BMD),^11^ which suggests that women with osteoporosis are at a higher risk of having cardiovascular events. Low BMD has been associated with an increased risk of echogenic calcified atherosclerotic plaque,^12^ and elderly women with echogenic carotid plaque are at a higher risk of nonvertebral fractures than are women without plaque.^13^ An association between lower BMD and structural and functional measurement of atherosclerosis (as assessed by the ankle-brachial index and IMT) has also been established in men.^14^ Standardization of carotid IMT measurements and recommendations for a carotid ultrasound scanning protocol were recently published,^14,15^ thereby enabling homogenous data collection and analysis.

## OBJECTIVE

The aims of the present study were to determine the relationship between IMT, presence of calcified atherosclerotic plaque and BMD measured at different sites, in postmenopausal women referred for routine bone density screening; and to establish whether there is any relationship between calcified atherosclerotic plaque in the carotid artery and osteoporotic vertebral fractures.

## MATERIAL AND METHOD

One hundred postmenopausal white women were consecutively enrolled from among patients presenting for BMD measurement in the Rheumatology Clinic of the "Iuliu Hatieganu" University of Medicine and Pharmacy, Cluj-Napoca, Romania, between September 2009 and February 2010. Women were eligible if menopause had occurred at least two years prior to their visit. Patients using hormone replacement therapy or medication affecting bone metabolism (corticosteroids, anticonvulsants or oral anticoagulants) were excluded. We also excluded subjects with any condition that might interfere with bone metabolism, such as thyroid disorders, malabsorption, chronic renal and liver diseases or alcoholism. None of the subjects had diseases (fractures, etc.) that interfered with their activities of normal daily life. In this group, 65 patients had hypertension, 35 patients had non-insulin-dependent diabetes and 28 had both diseases. Of these subjects, 31 were being treated with angiotensin-converting enzyme inhibitors, 25 with calcium antagonists, 15 with beta-blockers, four with angiotensin II type I receptor blockers, 19 with statins and two with fibrates.

Written informed consent for participation was obtained from each subject prior to enrolment. The Ethics Committee of our University approved the study protocol (ANCS 42107/2008 PNII grant). All the measurements were performed on the same day.

### Clinical assessments

Demographic and clinical variables were recorded, including age, weight, height, body mass index (BMI = weight/height^2^, kg/m^2^) and time elapsed since menopause. We also recorded the patients’ medication histories and associated diseases, including hypertension and diabetes. Their blood pressures were measured using a sphygmomanometer on the right arm of the subject, after 10 minutes of resting in the supine position.

### Assays

Blood samples were drawn from the antecubital vein in the morning after the subject had fasted for 12 hours. The serum concentrations of total cholesterol, triglycerides, creatinine and glucose were determined by means of standard laboratory techniques (Cobas Mira Plus analyzer).

### BMD measurements

BMD was measured in the lumbar spine (L2-L4) and femoral neck by means of dual energy X-ray absorptiometry (DXA), using the Lunar Prodigy Advance apparatus (GE Healthcare, United States). The same densitometer was used for all BMD measurements. The results were expressed as T-scores, i.e. the standard deviation (SD) from the peak adult BMD. The women were classified in three groups: normal BMD; osteopenia or low bone mass, i.e. a BMD value that was 1-2.5 SDs below the mean value for young adults; or osteoporosis, i.e. more than 2.5 SDs below the mean value for young adults (according to the World Health Organization's criteria for diagnosing osteoporosis).^16^ Three sites for BMD assessment were considered:^17,18^ lumbar spine (L2-L4), femoral neck and total hip. Because the total hip T-score values were greater than or equal to the femoral neck values in all cases, only the T-score values from the femoral neck were used in the statistical analysis. All the measurements were made by two operators. The inter- and intra-operator coefficients of variation were less than 1.3%.

To evaluate the prevalence of vertebral fractures, lateral radiographs (from T4 to L5) were used. A vertebral fracture was defined as a reduction of ≥ 20% in the anterior, middle and/or posterior height of the vertebral body.^19^

### Carotid IMT measurements

The carotid artery wall thickness was measured bilaterally using an ultrasonograph (Aloka Prosound alpha 10, Tokyo, Japan) with a 7.5 to 13 MHz linear probe, performed by the same examiner. The patient was in the supine position. The common carotid arteries were scanned longitudinally. The image was focused on the posterior (far) wall. Perpendicularity between the ultrasound beam and the far wall ensured good viewing of the IMT as two parallel echoic lines (the lumen-intima interface and media-adventitia interface). In all cases, three measurements of the common carotid artery far wall were made: at 10, 15 and 20 mm proximally to the bifurcation. If there was any atherosclerotic plaque in the region of interest, the measurement was made outside of the edges of the plaque. For each subject, the mean value of the three measurements was considered to be the current wall thickness of the distal common artery. The mean value between the right and left carotid artery IMT was also taken into account for the study. The variability of the ultrasonographic measurements was assessed by making two measurements on 15 volunteers over a one-week period. The reproducibility of the IMT measurement was 10%.

Calcified atherosclerotic plaque was defined as high echogenic focal widening of the carotid wall with protrusion into the lumen, associated with posterior shadow. Plaque was recorded as present or absent on the common, internal and external carotid artery walls.

### Statistical analysis

The data were expressed as the mean ± SD and standard error (SE). The 95% confidence intervals for the means were calculated. The chi-square test or Fisher exact test was used to compare categorical data. The Pearson or Spearman correlation coefficient was used between quantitative variables, according to their distribution. Means were compared using the t-test, Anova (analysis of variance) test, Mann-Whitney test or Kruskal-Wallis test, depending on variable distribution. The Anova test was followed by Scheffe post-hoc analysis. The test for normal distribution was the Kolmogorov-Smirnov test.

Multiple linear regression analysis was used to examine the relationship between IMT as the dependent variable and BMI, age and T-scores for the lumbar spine (L2-L4) and femoral neck as the independent variables. The results from the multiple stepwise linear regression technique (only the significant variables for predictions of the dependent variable were entered in the model) were presented as non-standardized regression coefficients (b) with standard error, P-value and 95% confidence interval. The same multiple linear regression analysis was used with the T-score for the lumbar spine, the T-score for the femoral neck and the lowest T-score for the lumbar spine and femoral neck as the dependent variables and BMI, age and IMT as the independent variables.

Multiple logistic regression analysis was used to examine the likelihood of developing atherosclerotic plaques based on several independent variables: hypertension, diabetes, osteoporotic vertebral fractures, T-scores for the lumbar spine (L2-L4) and femoral neck, and IMT. The results from the multiple forward logistic regression technique (only the significant variables for predictions of the dependent variable were entered in the model) were presented as odds ratios (OR), 95% confidence intervals (95% CI) for odds ratios and P-values.

P ≤ 0.05 was considered statistically significant. The statistical analysis was performed using the Statistical Package for the Social Sciences (SPSS) software, version 13.00.

## RESULTS

The study group characteristics are summarized in Table 1. The mean age was 64.5 years and 18% were current smokers. It was found that 56% of the patients had unilateral or bilateral carotid calcified atherosclerotic plaque, 13% had osteoporotic vertebral fractures (as seen radiographically).

**Table 1.** Characteristics of the study group (100 patients)

				95% CI			
	Mean	SD	SE	Lower boundary	Upper boundary	Minimum	Maximum
Age (years)	64.80	10.36	1.04	62.75	66.85	46	89
Time elapsed since menopause (years)	17.50	10.61	1.06	15.40	19.60	2	40
BMI (kg/m^2^)	28.86	5.12	0.51	27.84	29.87	16	41.23
IMT of the right CCA (mm)	0.097	0.022	0.002	0.093	0.101	0.05	0.150
IMT of the left CCA (mm)	0.099	0.025	0.003	0.094	0.104	0.05	0.170
Mean IMT (mm)	0.098	0.022	0.002	0.094	0.102	0.05	0.145
Total cholesterol (mg %)	215.27	52.16	5.22	204.92	225.62	131	439
Triglycerides (mg %)	137.97	92.09	9.21	119.70	156.24	47	615
Glucose (mg %)	115.43	38.86	3.89	107.72	123.14	65	259
Lumbar spine BMD (g/cm^2^)	1.01	0.20	0.02	0.96	1.05	0.64	1.62
Lumbar spine T score	-1.52	1.60	0.16	-1.84	-1.20	-4.70	3.50
Lumbar spine Z score	-0.27	1.67	0.19	-0.64	0.11	-3.20	4.30
Femoral neck BMD (g/cm^2^)	0.84	0.14	0.02	0.81	0.88	0.54	1.38
Femoral neck T score	-1.08	1.33	0.13	-1.35	-0.82	-3.50	4.30
Femoral neck Z score	-0.02	0.95	0.11	-0.23	0.20	-2.20	3.50
Total hip BMD (g/cm^2^)	0.90	0.16	0.02	0.86	0.94	0.57	1.54
Total hip T score	-0.85	1.25	0.14	-1.13	-0.57	-3.50	4.30
Total hip Z score	0.34	1.17	0.13	0.08	0.61	-2.10	5.00

IMT = intima-media thickness; BMI = body mass index; CCA = common carotid artery; SD = standard deviation; SE = standard error; CI = confidence interval.

The characteristics of the study subjects assigned to the normal, osteopenic and osteoporotic groups using T-score values for the lumbar spine (L2-L4) are summarized in Table 2. There were significant differences between the osteoporotic and osteopenic groups (P < 0.05) and between the osteopenic and normal groups with regard to age (P < 0.05), but the T-score was not significantly correlated with age (r = −0.17, P = 0.10). There was a significance difference in relation to time elapsed since the menopause only when comparing the osteoporotic group with the normal group (P < 0.01), and the T-score was significantly correlated with this period of time (r = −0.24, P = 0.02). There was no significant different regarding BMI in the three groups, and no correlation with T-score was found (r = 0.18, P = 0.07). IMT in the right CCA (common carotid artery), IMT in the left CCA or mean IMT was significantly associated with T-score in the osteoporotic group, in comparison with the other two groups (P < 0.05). The carotid IMT was also significantly negatively correlated with the T-score (r = −0.26, P = 0.01 for IMT in the right CCA; r = −0.34, P = 0.001 for IMT in the left CCA; and r = −0.32, P = 0.001 for mean IMT). No significant P values were obtained in relation to the presence or absence of atherosclerotic plaque in the three groups.

**Table 2.** Characteristics of the study subjects assigned to the normal, osteopenic and osteoporotic groups using the T-score values for the lumbar spine (L2-L4)

	Normal n = 36	Osteopenia n = 32	Osteoporosis n = 32	P value
Age (years)	62.75 ± 9.76	63.03 ± 9.98*	68.88 ± 10.50†	0.03
Time elapsed since menopause (years)	13.83 ± 9.27	16.59 ± 11.58	22.53 ± 9.27†	0.002
BMI (kg/m^2^)	29.52 ± 4.93	29.20 ± 4.81	27.77 ± 5.59	0.34
IMT of the right CCA (mm)	0.093 ± 0.024	0.092 ± 0.022	0.106 ± 0.016*,†	0.01
IMT of the left CCA (mm)	0.093 ± 0.026	0.093 ± 0.022	0.113 ± 0.023‡,§	0.001
Mean IMT (mm)	0.093 ± 0.024	0.092 ± 0.021	0.110 ± 0.018‡,§	0.001
Atherosclerotic plaque (present/absent)	17/19	20/12	19/13	0.402

* P < 0.05 versus normal,

† P < 0.05 versus osteopenia,

‡ P < 0.01 versus normal,

§ P < 0.01 versus osteopenia.

IMT = intima-media thickness; BMI = body mass index; CCA = common carotid artery.

The characteristics of the study subjects assigned to the normal, osteopenic and osteoporotic groups using the T-score values for the femoral neck are summarized in Table 3. Significant differences in age were found between the groups (P < 0.05 for osteopenia versus normal; and P < 0.01 for osteoporosis versus normal) and the T-score had a significantly negative correlation with age (r = −0.40, P < 0.001). The time elapsed since the menopause had a significantly negative correlation with the T-score (r = −0.46, P < 0.001) and significant differences between the osteopenia and normal groups (P < 0.01) and between the osteoporosis and normal groups (P < 0.01) were established. There was a significant difference in BMI only between the osteopenic and normal groups (P < 0.05), but there was no significant correlation with the T-score (r = 0.20, P = 0.04). IMT in the right CCA, IMT in the left CCA or mean IMT was significantly associated with the T-score in the osteoporotic group, in comparison with the osteopenic group (P < 0.05) and normal group (P < 0.01 for IMT in right CCA and mean IMT and P < 0.05 for IMT in left CCA). The carotid IMT was significantly negatively correlated with the T-score (r = −0.36, P < 0.001 for the right CCA; r = −0.31, P = 0.001 for the left CCA; and r = −0.35, P < 0.001 for mean IMT). No significant P values were obtained in relation to the presence or absence of atherosclerotic plaque in the three groups.

**Table 3.** Characteristics of the study subjects assigned to the normal, osteopenic and osteoporotic groups using the T-score values for the femoral neck

	Normaln = 35	Osteopenia n = 50	Osteoporosis n = 15	P value
Age (years)	60.26 ± 7.35	65.78 ± 10.49*	72.13 ± 11.38†	0.001
Time elapsed since menopause (years)	11.77 ± 7.95	19.42 ± 10.77†	24.47 ± 9.43†	0.00005
BMI (kg/m^2^)	30.52 ± 5.07	27.67 ± 4.88*	28.93 ± 5.28	0.04
IMT of the right CCA (mm)	0.090 ± 0.022	0.097 ± 0.020	0.113 ± 0.018†,‡	0.003
IMT of the left CCA (mm)	0.092 ± 0.023	0.099 ± 0.026	0.115 ± 0.022*,‡	0.02
Mean IMT (mm)	0.091 ± 0.021	0.098 ± 0.022	0.114 ± 0.018†,‡	0.004
Atherosclerotic plaque (present/absent)	16/19	31/19	9/6	0.312

* P < 0.05 versus normal,

† P < 0.01 versus normal,

‡ P < 0.05 versus osteopenia.

IMT = intima-media thickness; BMI = body mass index; CCA = common carotid artery.

Taking into account the lowest values of the T-scores for the lumbar spine (L2-L4) and femoral neck (Table 4), there was a significant difference in the patients’ ages between the osteopenic and normal groups (P < 0.01). The Pearson correlation between T-score and age revealed a significant correlation (r = −0.32, P = 0.001). The time elapsed since the menopause was significantly different between the osteoporotic and osteopenic groups (P < 0.05) and between the osteoporotic and normal groups (P < 0.01). In the latter case, a significant negative correlation with T-score was obtained (r = −0.37, P = 0.000). There was no significant difference (P = 0.30) and no correlation (r = 0.18, P = 0.07) in the relationship with BMI. There was a significant difference in mean IMT between the osteoporotic and normal groups (P < 0.01) and between the osteopenic and normal groups (P < 0.05). The T-score was significantly negatively correlated with mean IMT (r = −0.33, P = 0.001).

**Table 4.** Characteristics of the study subjects assigned to the normal, osteopenic and osteoporotic groups using the lowest T-score for the lumbar spine (L2-L4) and femoral neck

	Normal n = 19	Osteopenia n = 45	Osteoporosis n = 36	P value
Age (years)	59.47 ± 6.56	63.60 ± 10.36	69.11 ± 69.11*	0.002
Time elapsed since menopause (years)	10.74 ± 6.56	15.96 ± 11.01	23.00 ± 9.21*,†	0.00005
BMI (kg/m^2^)	30.31 ± 4.56	28.88 ± 5.04	28.06 ± 5.46	0.30
Mean IMT (mm)	0.092 ± 0.025	0.091 ± 0.021	0.109 ± 0.017*,†	0.0004
Atherosclerotic plaque (present/absent)	7/12	27/18	22/14	0.17

* P < 0.01 versus normal,

† P < 0.05 versus osteopenia.

IMT = intima-media thickness; BMI = body mass index.

Multiple linear regression analysis (stepwise linear regression) revealed that mean IMT (OR: −23.92; 95% CI: −37.42 to −10.42; P < 0.001) and BMI (OR: 0.06; 95% CI: 0.01 to 0.12; P = 0.03) were associated with the T-score for the lumbar spine; age (OR: −0.05; 95% CI: −0.07 to −0.03; P < 0.001) was correlated with the T-score for the femoral neck; and mean IMT (OR: −20.23; 95% CI: −31.26 to −9.20; P < 0.001) and BMI (OR: 0.053; 95% CI: 0.005 to 0.10; P = 0.03) significantly predicted the lowest T-score for the lumbar spine (L2-L4) and femoral neck. Mean IMT was dependent on age (OR: 0.001; 95% CI: 0.001 to 0.001; P < 0.001) and T-score for the lumbar spine (OR: 0.003; 95% CI: −0.006 to −0.001; P = 0.003).

Atherosclerotic plaque was significantly associated with age (P < 0.001), time elapsed since menopause (P < 0.001) and mean IMT (P < 0.001). No significant P value was obtained in association with the T-score for the lumbar spine or femoral neck, or with total cholesterol and triglyceride levels (Table 5). From multiple logistic regression to examine the likelihood of developing atherosclerotic plaque based on several independent variables (hypertension, diabetes, osteoporotic vertebral fractures, T-score for the lumbar spine L2-L4 and femoral neck, and IMT), we found that the mean IMT (P < 0.001, OR 4.96E+26, E = 3.38 × 10^+25^), high blood pressure (P = 0.005, OR 4.54) and osteoporotic vertebral fractures (P = 0.048, OR 0.21) presented statistical significance.

**Table 5.** Characteristics of study groups depending on the presence or absence of carotid atherosclerotic plaque

	Atherosclerotic plaque	n	Mean	SD	SE	95% CI		P value
						Lower boundary	Upper boundary	Lower boundary
Age (years)	Absent	44	60.30	9.34	1.41	57.46	63.13	< 0.001
	Present	56	68.34	9.79	1.31	65.72	70.96	
Time since menopause (years)	Absent	44	13.02	10.15	1.53	9.94	16.11	< 0.001
	Present	56	21.02	9.66	1.29	18.43	23.61	
BMI (kg/m^2^)	Absent	44	27.72	5.08	0.77	26.18	29.27	0.05
	Present	56	29.75	5.02	0.67	28.40	31.09	
Mean IMT (mm)	Absent	44	0.09	0.02	0.00	0.08	0.09	< 0.001
	Present	56	0.11	0.02	0.00	0.10	0.11	
T-score for the lumbar spine (L2-L4)	Absent	44	-1.38	1.70	0.26	-1.90	-0.86	0.36
	Present	56	-1.63	1.53	0.20	-2.04	-1.22	
T-score for the femoral neck	Absent	44	-0.75	1.53	0.23	-1.22	-0.29	0.07
	Present	56	-1.34	1.09	0.15	-1.63	-1.05	
Total cholesterol (mg %)	Absent	44	209.16	55.18	8.32	192.38	225.94	0.11
	Present	56	220.07	49.63	6.63	206.78	233.36	
Triglycerides (mg %)	Absent	44	136.00	105.28	15.87	103.99	168.01	0.38
	Present	56	139.52	81.20	10.85	117.77	161.26	

IMT = intima-media thickness; BMI = body mass index; SD = standard deviation; SE = standard error; CI = confidence interval.

## DISCUSSION

In the present study, we showed that carotid IMT was greater in postmenopausal women with osteoporosis than in those with low bone mass or normal bone mass. At the same time, we showed that the association between IMT and BMD was independent of the sites of IMT measurement (right, left or mean CCA) and BMD measurement (T-scores for L2-L4 and femoral neck, and lowest T-score value at these sites). We also found that the presence of osteoporotic vertebral fractures acted as an independent risk factor for development of atherosclerotic plaque in carotid arteries.

Since 1986, when Pignoli et al.^20^ described a double line at the inner wall of the carotid artery in B-mode ultrasound, which was identified histologically as the intima and media layers of the vessel, numerous extensive studies have demonstrated a correlation between IMT and the presence of systemic atherosclerosis.^8,9,21-23^ These two lines represent the lumen-intima and media-adventitia interfaces. There is currently a slight disagreement regarding the location at which the measurements should be taken, but recommendations for carotid ultrasound scanning protocols^14,15^ were published recently in order to improve the power of clinical studies. Because of the lack of systems for semi-manual, automated readings^15^ or radiofrequency multiple M-line analysis,^24^ which could provide the mean value of IMT in a very short time, the method used in our study for IMT measurement followed the consensus statements already mentioned above.

To interpret the T-scores using DXA measurements, the World Health Organization's criteria for diagnosing osteoporosis^16^ were used. Because the measurements were made at multiple skeletal sites, analysis of the recorded data using the lowest T-score for the lumbar spine L2-L4, femoral neck and total hip was also performed.^25^ Interestingly, no value obtained from the total hip measurement was lower than the values from the femoral neck, so in fact, the lowest T-score was chosen between the lumbar spine and femoral neck T-score values.

The observed association between low bone mineral density and IMT is consistent with previous studies. Hmamouchi et al.^26^ reported a negative correlation between BMD and IMT in postmenopausal Moroccan women, independent of confounding factors, thus suggesting that there is a need for bone status evaluation in patients with vascular diseases. Tamaki et al.^27^ found that low spinal bone mass or presence of vertebral fractures were independently associated with increased IMT at the carotid bifurcation among women in their first 10 postmenopausal years. Uyama et al.^28^ indicated that there was an association between atherosclerosis and BMD for the total body, but not the spine.

The sites used for BMD measurement by means of DXA have differed: lumbar spine,^10,29,30^ distal and ultradistal radius,^12^ lumbar spine and total hip,^11,27^ and lumbar spine and all hip sites.^26^ In some studies, an opinion was formed that there was an independent association between BMD at cortical sites and vascular calcification,^26,28^ while in others the relationships between cortical/trabecular bone and carotid artery plaque were shown to be unconnected to the measurement sites.^11,12^ In our study, we used the lumbar spine T-score (trabecular bone), the femoral neck T-score (cortical bone) and the lowest T-score of these sites, for comparisons. In all these situations, IMT in the right CCA or left CCA or the mean IMT was significantly associated with the T-score in osteoporotic patients, in comparison with the normal and osteopenic groups. Also, the T-score had a significant negative correlation with the mean IMT. Our results suggested that the association between atherosclerosis and osteoporosis was independent of the sites for DXA T-score and CCA IMT determinations.

Our multiple logistic regression analysis on the presence of atherosclerotic plaque in relation to T-scores showed that there was a statistically significant likelihood that osteoporotic vertebral fractures might develop. The association between calcified atherosclerotic plaque and BMD was previous confirmed in other studies,^11,12,26,31^ which suggests that calcification of the atherosclerotic plaque may be a regulated process similar to that of osteogenesis. Some recent studies have implicated osteoprotegerin (an indirect inhibitor of osteoclastogenesis and indicator of bone remodeling) in the process of human atherogenesis.^32,33^ It is unclear whether osteoprotegerin should be considered to be an independent risk factor for cardiovascular diseases^11^ or to be a defense mechanism for atherosclerotic progression.^30^

There are several limitations to our study. The first limitation relates to its cross-sectional design, which implies that no causal inferences can be drawn. The study group was selected from among patients who had undergone DXA and not from the community, thus explaining the relatively high incidence of osteoporosis. Secondly, the presence of atherosclerotic plaque was correlated only with total cholesterol, and not with LDL-cholesterol. Finally, we did not quantify the atherosclerotic plaque and we did not take into account the anti-atherosclerotic effects of antihypertensive lipid- and glucose-lowering drugs.

## CONCLUSIONS

In conclusion, among women referred for routine osteoporosis screening, the relationship between atherosclerosis (as assessed by IMT and calcified plaque in carotid arteries) and osteoporosis (assessed by DXA) can be demonstrated. This relationship is valuable for comparisons between either cortical or trabecular bone and the left, right or mean CCA IMT. There was also a statistically significant relationship between the presence of calcified atherosclerotic plaque and osteoporotic vertebral fractures. These findings support the hypothesis that there are common pathological pathways involved in these two diseases.
